# Positive selection in octopus haemocyanin indicates functional links to temperature adaptation

**DOI:** 10.1186/s12862-015-0411-4

**Published:** 2015-07-05

**Authors:** Michael Oellermann, Jan M. Strugnell, Bernhard Lieb, Felix C. Mark

**Affiliations:** Integrative Ecophysiology, Alfred-Wegener-Institute Helmholtz Centre for Polar and Marine Research, Am Handelshafen 12, 27570 Bremerhaven, Germany; Department of Genetics, La Trobe Institute for Molecular Sciences, La Trobe University, Bundoora, VIC 3086 Australia; Institute of Zoology, Johannes Gutenberg-Universität, Müllerweg 6, 55099 Mainz, Germany

**Keywords:** Cephalopoda, oxygen binding, net surface charge, cold tolerance

## Abstract

**Background:**

Octopods have successfully colonised the world’s oceans from the tropics to the poles. Yet, successful persistence in these habitats has required adaptations of their advanced physiological apparatus to compensate impaired oxygen supply. Their oxygen transporter haemocyanin plays a major role in cold tolerance and accordingly has undergone functional modifications to sustain oxygen release at sub-zero temperatures. However, it remains unknown how molecular properties evolved to explain the observed functional adaptations. We thus aimed to assess whether natural selection affected molecular and structural properties of haemocyanin that explains temperature adaptation in octopods.

**Results:**

Analysis of 239 partial sequences of the haemocyanin functional units (FU) f and g of 28 octopod species of polar, temperate, subtropical and tropical origin revealed natural selection was acting primarily on charge properties of surface residues. Polar octopods contained haemocyanins with higher net surface charge due to decreased glutamic acid content and higher numbers of basic amino acids. Within the analysed partial sequences, positive selection was present at site 2545, positioned between the active copper binding centre and the FU g surface. At this site, methionine was the dominant amino acid in polar octopods and leucine was dominant in tropical octopods. Sites directly involved in oxygen binding or quaternary interactions were highly conserved within the analysed sequence.

**Conclusions:**

This study has provided the first insight into molecular and structural mechanisms that have enabled octopods to sustain oxygen supply from polar to tropical conditions. Our findings imply modulation of oxygen binding via charge-charge interaction at the protein surface, which stabilize quaternary interactions among functional units to reduce detrimental effects of high pH on venous oxygen release. Of the observed partial haemocyanin sequence, residue 2545 formed a close link between the FU g surface and the active centre, suggesting a role as allosteric binding site. The prevalence of methionine at this site in polar octopods, implies regulation of oxygen affinity via increased sensitivity to allosteric metal binding. High sequence conservation of sites directly involved in oxygen binding indicates that functional modifications of octopod haemocyanin rather occur via more subtle mechanisms, as observed in this study.

**Electronic supplementary material:**

The online version of this article (doi:10.1186/s12862-015-0411-4) contains supplementary material, which is available to authorized users.

## Background

For over 400 million years [1–3], cephalopods have been vital members of ecosystems spanning tropical to polar regions [4, 5]. Cephalopods play an important role as both consumers and prey [6] and there are about 800 known extant species [6, 7]. Despite the fact they are molluscs, cephalopods often directly compete with fishes [8, 9]. This is facilitated not only by the possession of a highly developed nervous system but particularly due to their advanced oxygen supply system. The cephalopod oxygen supply system is powered by three hearts, which push blood containing the oxygen carrier haemocyanin through their closed circulatory system [10–12].

As ectotherms, cephalopods are required to sustain metabolic performance and thus body functions at their respective habitat temperature. This task seems challenging considering the large temperature variation in temperate climates or the sub-zero temperatures prevailing in polar waters. Temperature is known to affect oxygen supply in marine ectotherms and even to cause the failure of oxygen supply at the margins of an animal’s thermal niche [13, 14]. This has also been confirmed in cephalopods. In the common cuttlefish, *Sepia officinalis*, blood perfusion failed to fuel the increased metabolic demand for oxygen at higher temperatures [15]. At lower temperatures however, recent evidence ascribes haemocyanin a major role in limiting cold tolerance [15, 16]. This is because the affinity of haemocyanin for oxygen increases as temperature decreases, an effect which decreases oxygen release to the tissues in *Sepia officinalis* and octopods at lower temperatures. However, the Antarctic octopod, *Pareledone charcoti*, was shown to mitigate this detrimental effect by an increased expression of a haemocyanin with lowered affinity for oxygen, and an increased rate of pH dependent oxygen release. This allows haemocyanin of *Pareledone charcoti* to release oxygen at higher pH than haemocyanins from warm water octopods [16].

While much progress has been made in understanding the underlying molecular and evolutionary mechanisms that explain adaptations of blood oxygen transport in polar fishes [17, 18], such mechanisms remain unknown in polar ectotherms, which use haemocyanin as blood oxygen carrier. This is surprising, as cephalopod haemocyanins have been considered among the best understood of the molluscan respiratory proteins for over 20 years [19–21]. Between ~3.4-4.0 MDa in size, cephalopod haemocyanins are among the largest known respiratory pigments [22, 23]. They form a single cylinder built from ten subunits, each composed of a ‘chain’ of eight (in decapods such as squids and cuttlefish) or seven (in octopods and *Nautilus spp.*) paralogous functional units (FU) termed as FU a, b, c, d, (d*,) e, f, g (d* indicates the duplicated FU d found in decapod cephalopods [21–23]). In octopods and *Nautilus*, subunits are 350 kDa in size, with FU a-f forming the wall structure of the cylindrical haemocyanin decamer and FU g a collar like structure at the inside of the cylinder (for structural details see [21, 22]). Every FU binds one dioxygen molecule to a central pair of copper atoms, each of which is coordinated by three histidines, enabling each octopus haemocyanin to carry up to 70 oxygen molecules [24]. Recurring duplication events of one subunit coding gene has led to the presence of two or even three haemocyanin isoforms in cephalopods [23, 25].

Despite the well-established structural details it remains unknown how molecular and structural features evolved to enable haemocyanin mediated oxygen supply in cephalopods at temperatures as low as −1.9 °C. We therefore aimed to 1) assess whether natural selection affected the haemocyanin gene and if so, 2) how selection on particular sites or regions may have affected haemocyanin function and 3) lastly, if this explains mechanisms of cold adaptation previously observed in octopods [12, 16, 26].

In this study we analysed two partial regions of the haemocyanin gene, spanning both wall- and collar-type functional units and compared them among 28 species of polar, temperate, subtropical and tropical benthic octopods. In this sequence of 396 amino acids, we found 13 sites under positive selection (3.3 %) predominantly at the FU surface suggesting functional modifications via indirect charge-charge interactions that stabilize quaternary interaction among FUs in cold-adapted octopods. Additional analysis of the entire haemocyanin gene of the Antarctic octopus *Pareledone charcoti* suggests that these findings apply to other FUs as well. The presence of a potential allosteric site in FU g may further support oxygen release in the cold.

## Results

### Phylogenetic analysis

**Fig. 1:**
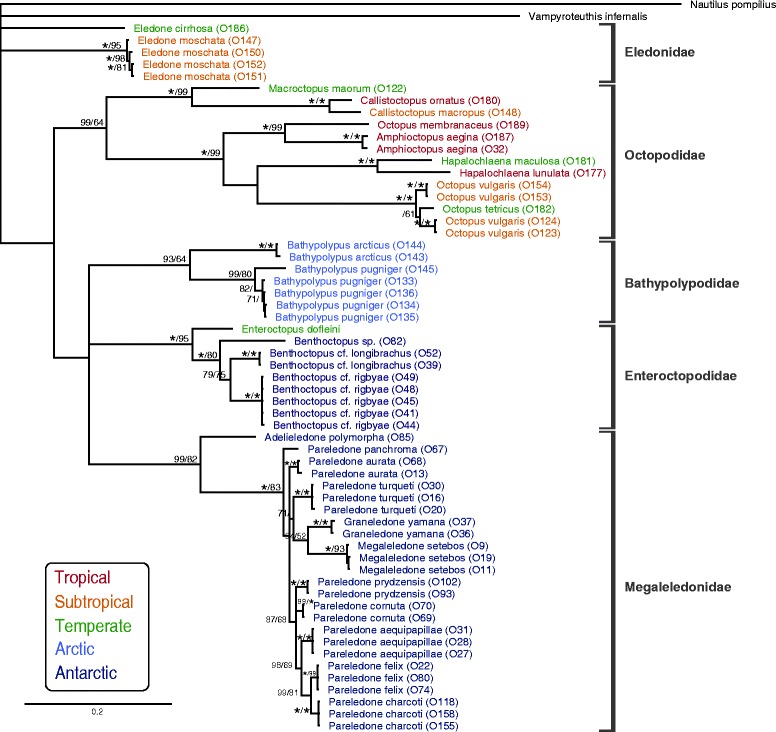
Bayesian phylogenetic tree illustrating species and family relationships among the analysed octopods. Bayesian analysis was performed on concatenated data sets of the mitochondrial genes cytochrome oxidase subunit I (COI) and cytochrome oxidase subunit III (COIII). Colours represent the climatic origin. Nodes were labelled with posterior probabilities and maximum likelihood bootstrap support values following a backslash, above a threshold of 0.7 or 0.5 respectively. Asterisks mark values of 100. Identical colours mark octopods of the same climatic origin. Sample codes are indicated in brackets.

In this study we analysed 59 individuals of 28 benthic octopods species. New sequences obtained in this study included 57 sequences of cytochrome c oxidase subunit I (COI), 57 sequences of cytochrome c oxidase subunit III (COIII) as well as 239 partial haemocyanin sequences (Additional file 1 ). COI and COIII have been frequently employed to resolve species relationships among cephalopods elsewhere [e.g. 27–29] and were thus used as a ‘phylogenetic standard’ in comparison to the haemocyanin phylogeny to identify cues of natural selection. Phylogenetic relationships resulting from Bayesian and maximum likelihood (ML) analyses of COI and COIII sequence data conformed to the most recent classification of octopod families (Fig. 5 in [27]). The analysed Antarctic octopods included two families; 11 species of the Megaleledonidae sampled in sub-Antarctic and Antarctic waters as well as three species of the Enteroctopodidae exclusively sampled in sub-Antarctic waters (for sample details see Addtional file 2 & Additional file 3). Arctic octopods comprised two species of the Bathypolypodidae. Non-polar octopods comprised 12 species; five temperate species represented in the Enteroctopodidae, Eledonidae and the Octopodidae, three subtropical species represented in the Eledonidae and the Octopodidae and four tropical species found in the Octopodidae only (Fig. 1).

**Fig. 2:**
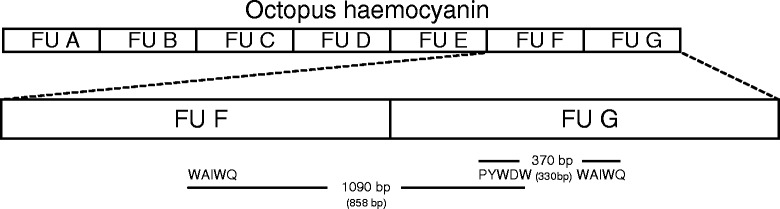
Schematic illustration of the functional units of the octopus haemocyanin gene and the partial regions analysed in this study. Conserved binding sites of the degenerate forward and reverse primers are marked with the corresponding amino acid sequence WAIWQ or PYWDW. Resulting amplicons spanned a region between the functional units f and g and a region within the functional unit g. Fragment lengths are indicated before (in line) and after sequence trimming (in parentheses).

**Fig. 3:**
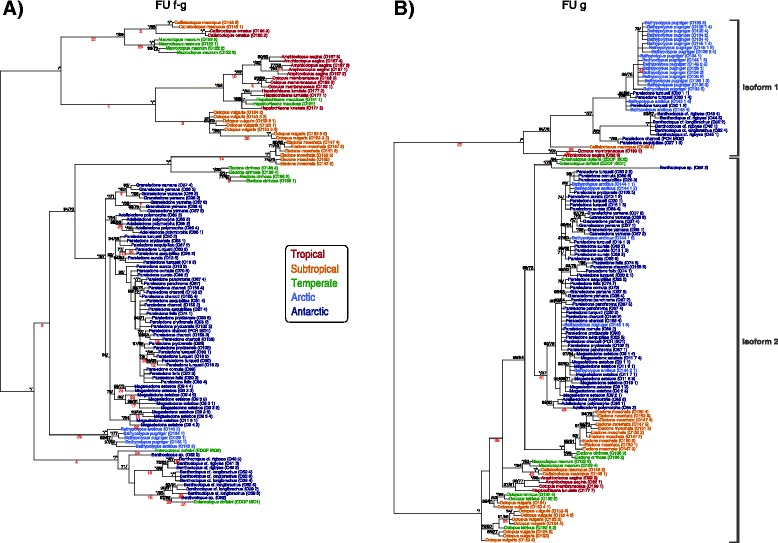
Haemocyanin based phylogenetic relationships among the analysed octopods. Bayesian phylogenetic trees based on the nucleotide alignments of **a**) a partial haemocyanin region between FU f and FU g **b**) a partial haemocyanin region within FU g (see Fig. 2). Note that within FU g two distinct isoforms were present causing a split in the phylogeny. Nodes were labelled with posterior probabilities and maximum likelihood bootstrap support values following a backslash, above a threshold of 0.7 or 0.5 respectively. Asterisks mark values of 100. Red italic numbers below branches indicate positive selection of a particular site at this branch inferred from TreeSAAP analysis (refer to Table 1). Identical colours mark octopods of the same climatic origin. Sample codes are indicated in brackets and followed by the clone identifier.

Two separate phylogenies of the haemocyanin gene based on a 1) 858 bp long region spanning FU f to FU g and a 2) 330 bp long region within the FU g (Fig. 2) had some important topological differences to the recent octopod classification [27]. For the haemocyanin region FU f-g, all octopod families identified with COI and COIII formed distinct clades as well, yet with the Eledonidae being a well-supported sister group to the Antarctic Megaleledonidae (Fig. 3a), which was not the case in the COI/COIII phylogeny (Fig. 1). For the haemocyanin region FU g, neither the Antarctic Megaleledonidae nor the Arctic Bathypolypodidae formed monophyletic groups, rather species within these two families were mixed together within a single clade (Fig. 3b), despite being distinct families in the COI-COIII phylogeny (Fig. 1). Octopodidae species also did not form a well-supported monophyletic group (Fig. 3b). The Eledonidae and Enteroctopodidae remained distinct, with the latter forming a well-supported sister group with the polar Megaleledonidae and Bathypolypodidae (Fig. 3b). However, within the COI-COIII phylogeny such relationship could not be resolved with these three clades forming a polytomy (Fig. 1).

The analysis retrieved at least two isoforms for most families that, however, were often not easily discernible from allelic variation. Most apparently, in the partial haemocyanin region FU g two distinct isoforms were present, contained in both octopods from warm and cold climates that caused a major split of the phylogeny (Fig. 3b). This was at least partly due to the lack of a five amino acid long region (i.e. DPEKG indel) at positions 2638–2643 (positions refer to the full haemocyanin sequence of *Enteroctopus dofleini*, [GenBank: O61363]) in the FU g isoform 1, which was most frequent in the Bathypolypodidae and the Antarctic Enteroctopodidae (Fig. 3b, Additional file 4).

### Natural selection in octopus haemocyanin

**Fig. 4:**
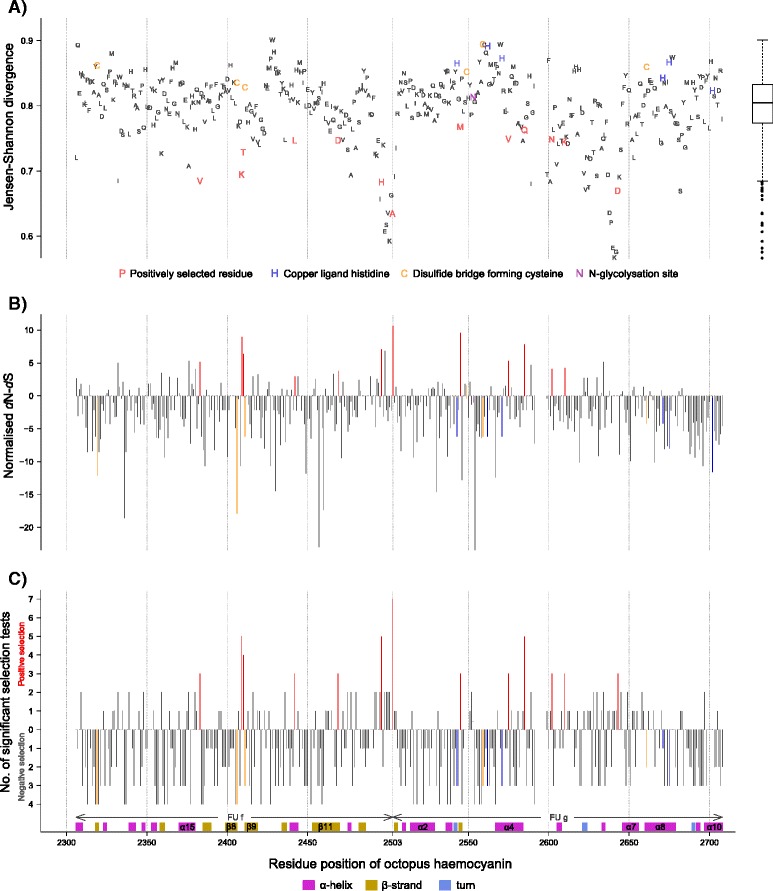
**a**) Protein sequence conservation estimated using the Jensen-Shannon divergence [30], which identifies conserved sites as deviations of a probability distribution from the overall amino acid distribution of the respective BLOSUM62 alignment as background and also accounts for conservation in neighbouring sites. Greater scores indicate higher sequence conservation. **b**) Estimated differences between nonsynonymous and synonymous substitutions (*d*N-*d*S) at each site of the haemocyanin nucleotide sequence inferred from Single Likelihood Ancestral Counting (SLAC) normalised to the total length of the underlying maximum likelihood tree. **c**) Natural selection in octopus haemocyanin. The y-axis indicates the cumulative number of selection tests that yielded significant results for positive or negative selection at an individual site. Positively selected sites confirmed by two or more tests were marked red. All analysis was performed for two separate alignments, containing 113 or 126 sequences respectively, and covering in total a 396 amino acid long coding region of the haemocyanin’s functional units f and g. Residue positions refer to the published full haemocyanin sequence of *Enteroctopus dofleini* [UniProt: O61363] [23, 24]. Significance thresholds were: p-values ≤ 0.10 for SLAC, ≤ 0.10 for FEL, MEME and PRIME; Posterior Probability ≥ 0.90 for FUBAR; Bayes Factor ≥ 0.50 for EF.

The analysed partial haemocyanin regions of FU f-g and FU g spanning the amino acid positions 2306–2708 were highly conserved, as indicated by the Jensen-Shannon Divergence measure (JSD, [30]) being 0.80 on average (95 % C.I. 0.790-0.801, *n* = 396) and larger than 0.73 for more than 90 % of positions (Fig. 4a). Particularly sites known to be involved in oxygen binding or protein stability were highly conserved such as the copper binding histidines (JSD 0.82-0.89), disulfide or thioether bridge forming cysteines (JSD 0.83-0.89), or other residues within a 7 Å radius of the active site (Fig. 4a, [24]). Pronounced sequence variation was found within the peptide linking the FU f and FU g (positions 2495–2504) as well as at the region flanked by the alpha helix α6 and α7 (positions 2638–2644) located within a structural FU domain rich in α helices (Fig. 4a). Variation in the latter was mostly explained by the DPEKG indel at this position (Fig. 4a, Additional file 4).

Analysis of the partial haemocyanin regions revealed both negative as well as positive selection. While negative selection removes alleles that are detrimental to protein function and thus ‘purifies’ genes from sequence variability, positive selection increases the frequency of advantageous alleles. Negative selection prevailed within the analysed haemocyanin regions as indicated 1) by the high conservation of the haemocyanin genes (mean JSD 0.80), 2) predominant negative differences of non-synonymous and synonymous codon substitutions (65 % of sites with *d*N-*d*S < 0 vs. 23 % of sites with *d*N-*d*S > 0) inferred from Single Likelihood Ancestral Counting (SLAC, Fig. 4b) as well as 3) by codon based maximum likelihood and Bayesian analysis, which identified 26 % of sites being under significant negative selection (i.e. sites were considered significantly selected if at least three out of seven selection tests were significant at a site, Fig. 4c). In contrast, only 13 sites of the analysed 396 codons (3.3 %) were identified to be positively selected (Table 1, Fig. 4c). Amino acid properties being affected at these positively selected sites included the isoelectric point identified by both PRIME and TreeSAAP analysis, as well as chemical composition (PRIME only), equilibrium constant (ionization of COOH), alpha helical tendencies and the power to be at the C-terminal (TreeSAAP only, Table 1).

**Table 1:** Positively selected sites in octopus haemocyanin

Residue	SLAC	FEL	MEME	FUBAR	EF	PRIME^a^				TreeSAAP^b^			
										pHi (8)	pK’ (8)	pα (6)	PCT (6)
2383		*	*								↓**(1)**		
	*										↑**(2–4)**		
2409													
2410											↓**(5–8)**		
2442						(CC)					↓**(10)** ↑**(5,9,11)**		
2469						**CC**						↓**(12,13)**	
2496										↓**(14,15)**			
2503						(P)						↓**(14,15,22-27)** ↑**(20)**	
2545											↓**(4, 20, 28–30)** ↑**(9,19,31,32)**		
2575												↓**(33,34)**	
2585						**pHi**	**CC**	(P)	(V)				
2602										↓**(35,36)**			↑**(35,36)**
2610						pHi				↓**(37–39)** ↑**(40,41)**			↓**(40–42)** ↑**(37–39)**
2643						CC							↓**(38)**

Sites at which at least three or more selection tests identified significant positive selection. Analysis was performed for two separate alignments, containing 113 or 126 sequences respectively, and covering in total a 396 amino acid long region of the haemocyanin’s functional units f and g. Numbering of positions refers to the published full haemocyanin sequence of *Enteroctopus dofleini* [UniProt: O61363] [23, 24]. Significance thresholds were: *P* ≤ 0.10 for SLAC, ≤ 0.10 for FEL, MEME and PRIME; Posterior Probability ≥ 0.90 for FUBAR; Bayes Factor ≥ 0.50 for EF. See Additional file 5: Table S5 for detailed results.

Significant test result ; CC Selection for this property; (CC) Conservation of this property

^a^CC Chemical Composition; *P* Polarity; *V* Volume; *pHi* Iso-electric point; *H* Hydropathy. ^b^ *pHi* isoelectric point; *pK’* equilibrium constant (ionization of COOH); *pα* alpha helical tendencies; *PCT* Power to be at the C-terminal (Magnitude of amino acid change)

### Structural links to protein function

**Fig. 5:**
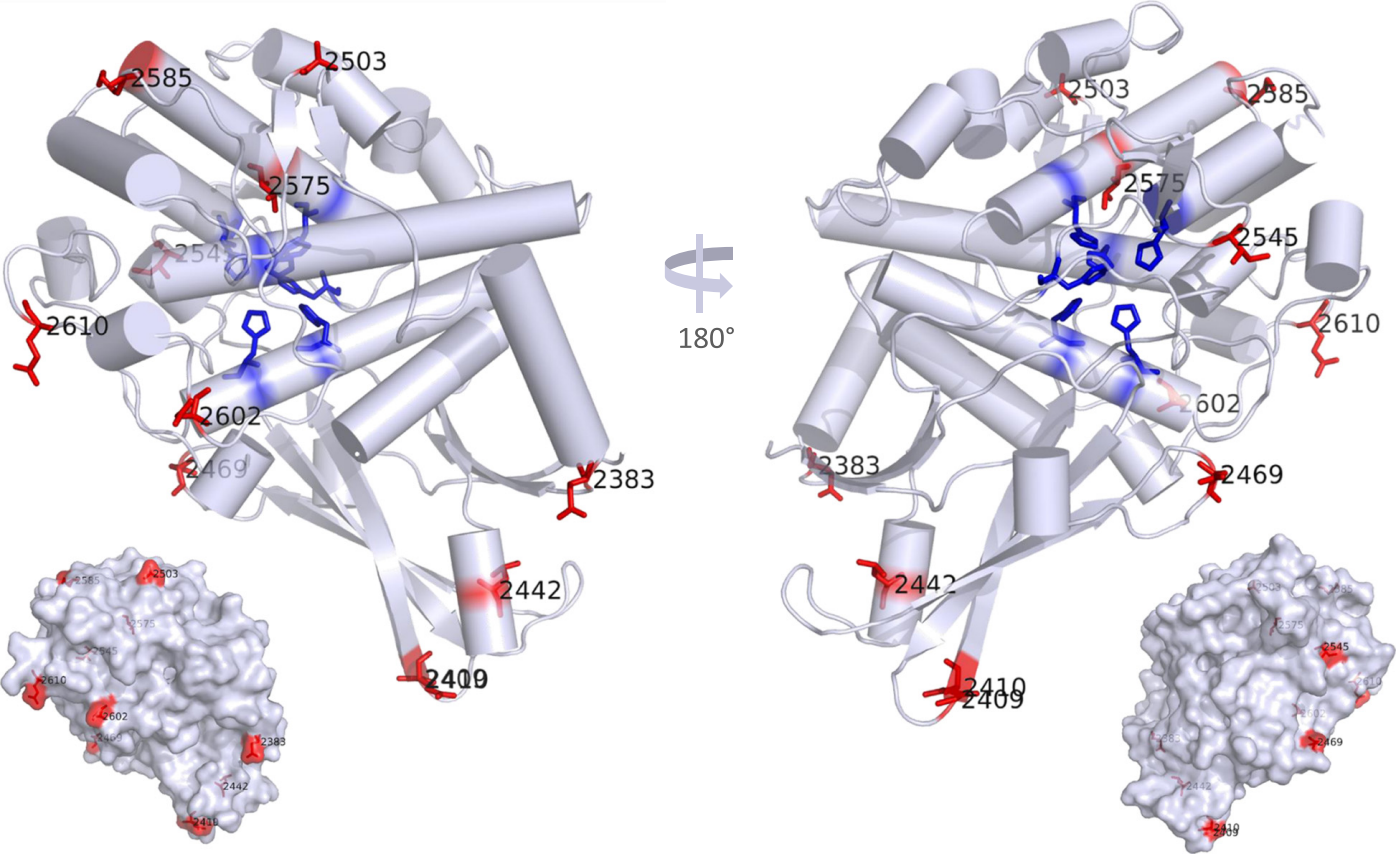
Steric arrangement of positively selected sites in octopus haemocyanin. The 3D model represents the haemocyanin molecule of the functional unit g of *Enteroctopus dofleini* ([PDB: 1JS8], [24]). Positively selected sites were marked red and labelled with its residue number. Copper ligand histidines were marked blue. Due to strong homology, positions of positively selected sites from functional unit f could be mapped onto the FU g 3D structure. 3D structures below illustrate positively selected sites located at the protein surface

Eleven out of the 13 positively selected sites could be mapped onto the published crystal structure of the haemocyanin FU g ([PDB: 1JS8], Fig. 5). Seven of these sites were located at the surface of FU g and FU f and only three sites at the inside. One site, located in FU g at position 2585, was embedded into a small surface pocket and therefore was neither defined as buried or exposed (Fig. 5).

The seven positively selected surface residues as well as the residue at position 2585, were largely characterized by changes of their side chain charge with either substitutions among polar amino acids as is the case for most sequences at positions 2602 and 2610, 2409 and 2585 (Table 1) or substitutions between polar and hydrophobic amino acids as is the case for most sequences at positions 2383, 2410, 2469 (Additional file 4). The site at position 2503 was located in the linker region between FU f and FU g and showed the by far highest number of substitution events; 18 non-synonymous substitutions, involving alanine, valine, threonine, or serine (Additional file 4). Changes of polar amino acids also affected the residue at position 2496 located at the end of FU f, however this residue could not be mapped onto the published haemocyanin crystal structure.

The buried residues at position 2545 and 2575 were only two or four residues adjacent to the copper binding histidines His2543 or His2571, respectively (Fig. 5, Additional file 4). Position 2545 was characterized by its proximity to both the copper binding His2543 and the FU surface. It was embedded in a surface pocket surrounded by highly conserved neighbours, comprising the hydrophobic Pro2546, Leu2547, Tyr2691 and the hydrophilic His2690 and His2834 as well as the variable residue 2622, which included valine in the FU g isoform 2 and polar serine and threonine in the FU g isoform 1 (Fig. 3b). At site 2545, three exclusively hydrophobic amino acids were present, methionine, isoleucine or leucine, whose side chains were > 7 Å distant from the closest copper binding histidine (His2543) (distances calculated from the haemocyanin FU g model, [PDB: 1JS8]). At position 2575, three different amino acids were present across the alignment; threonine, alanine and valine (Additional file 4), of which only threonine was able to form polar interaction. The analysis of the structural environment using PhyMol showed that the only polar neighbour in proximity of the polar -OH group was the terminal amino group of His2571 (2.8 Å), which however, is involved in a peptide bond with Arg2572. The amino groups of the histidine side chains were too distant for any polar interaction (≥5.6 Å, weak hydrogen bonding and salt bridge formation begins at ≤4.0 Å [31, 32]). The third buried residue 2442 was located within the alpha helix α16 and comprised exclusively aliphatic residues, methionine, isoleucine, leucine or valine (Additional file 4).

### Correlates of temperature adaptation

**Fig. 6:**
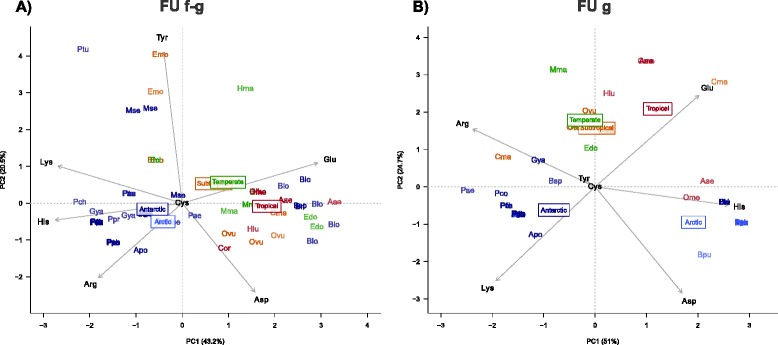
Principal component analysis for polar surface residues of octopus haemocyanin. The analysis was performed separately **a**) for 65 surface residues of the partial haemocyanin fragment FU f-g and **b**) for 19 surface residues of the partial haemocyanin fragment FU g. Surface residues were identified for the haemocyanin structural model [PDB: 1JS8] using GETAREA [97]. PCA variables comprised the total number of each type of polar surface residue as well as their total net charge. Identical colours mark octopods of the same climatic origin. Rectangles mark the centroid of the respective climatic group. PCA scores were abbreviated with species names as followed: *Apo* Adelieledone polymorpha, *Bar* Bathypolypus arcticus, *Blo* Benthoctopus longibrachus, *Bpu* Bathypolypus pugniger, *Bri* Benthoctopus rigbyae, *Bsp* Benthoctopus sp., *Cma* Callistoctopus macropus, *Cor* Callistoctopus ornatus, *Eci* Eledone cirrhosa, *Edo* Enteroctopus dofleini, *Emo* Eledone moschata, *Gya* Graneledone yamana, *Hlu* Hapalochlaena lunulata, *Hma* Hapalochlaena maculosa, *Mma* Macroctopus maorum, *Mse* Megaeledone setebos, *Ome* Octopus membranaceus, *Ovu* Octopus vulgaris, *Pae* Pareledone aequipillae, *Pau* Pareledone aurata, *Pch* Pareledone charcoti, *Pco* Pareledone cornuta, *Pfe* Pareledone felix, *Ppa* Pareledone panchroma, *Ppr* Pareledone prydzensis, *Ptu* Pareledone turqueti

Based on the predominant positive selection on charge properties of haemocyanin FU surface residues, we assessed the effect of climate origin of octopods on polar surface residues for each of the two haemocyanin regions. Principal component analysis, based on total counts of each polar amino acid type, showed a distinct separation of species from cold and warm climates, with tropical octopods being most distant from Antarctic and Arctic octopods. Sequences of temperate and subtropical octopods overlapped and occurred between those of polar and tropical octopods in the PCA plots with respect to their composition of charged amino acids (Fig. 6). The Antarctic Enteroctopodidae, however, did not group with the Antarctic Megaleledonidae but rather with octopods from warmer climates in (FU f-g, Fig. 6a) or within a distinct cluster formed by the isoform 1 present in FU g (Fig. 6b).

**Fig. 7:**
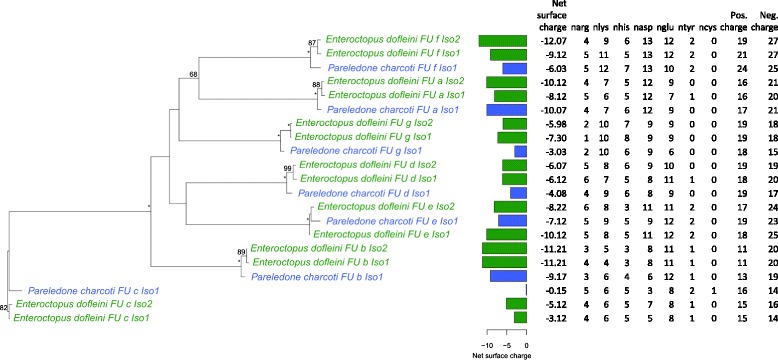
Net surface charges for haemocyanin FU a-g compared between the temperate North-Pacific octopus Enteroctopus dofleini and the Antarctic octopus Pareledone charcoti. Maximum likelihood phylogenetic tree based on the amino acid alignment of FU a-g of both species. Support values calculated from 1000 bootstrap replicates are given above nodes if larger than 50. Asterisks indicate values of 100. Total net charge and numbers of charged residues were calculated from 85 surface residues of each FU and presented in detail

The frequency of glutamic acid increased in both haemocyanin regions towards octopods from warmer climates, except for the Antarctic Enteroctopodidae, and correlated negatively with numbers of positively charged amino acids (His and Arg in FU f-g and Lys in FU g, Fig. 6), which accordingly increased by frequency in octopods from polar climates. Numbers of cysteine and tyrosine did not help to explain the divisions between climate origins in both FU regions. The observed pattern also persisted independently of evolutionary relationships. For example, temperate octopods from three distinct families grouped closely together (Fig. 6b). Similarly, Arctic octopods grouped within the Antarctic Megaleledonidae despite these being well understood to be distinct families (Fig. 6a). Due to the climate dependent trend of acidic or basic surface residues, the net charge of surface residues was highly correlated with climate in FU f-g (*P*_Kendall’s rank_ = <0.001, tau −0.386), however not in FU g (*P*_Kendall’s rank_ = 0.203, tau −0.097). The lack of correlation in FU g was due to the differing surface charge properties between the two distinct isoforms present in FU g (Fig. 3b). When analysed separately, isoform 2 conformed to the high correlation between climate origin and net charge (*P*_Kendall’s rank_ = <0.001, tau −0.494), yet not isoform 1, which showed a slightly positive trend between climate origin and net charge (*P*_Kendall’s rank_ = 0.040, tau 0.34). Separation between the two isoforms in FU g was mostly determined by a higher histidine and lower arginine content in isoform 1 (Fig. 6b). The analysis of entire haemocyanin sequences showed a more positive net surface charge across all FUs except FU a in the Antarctic octopus *Pareledone charcoti* compared to the temperate giant Pacific octopus *Enteroctopus dofleini* (Fig. 7), which confirms findings for the partial regions FU f-g and FU g (Fig. 6). Charge differences were due to both increasing numbers of positively charged and decreasing numbers of negatively charged amino acids (Fig. 7).

Moreover, residue 2545 varied between the three hydrophobic amino acids, of which methionine prevailed in polar octopods and leucine in tropical octopods. Of the 73 haemocyanin sequences from polar octopods 88 % contained methionine, 12 % isoleucine and 0 % leucine. In contrast, of the 13 haemocyanin sequences from tropical octopods, three sequences contained methionine (all exclusive to *H. lunulata*), none contained isoleucine and ten sequences contained leucine (three species). Subtropical and temperate octopods showed a mixed pattern for these amino acids (Additional file 4). Lastly, although residue 2503 was highly positively selected, there was no correlation with climatic origin (*P*_Kendall’s rank_ = 0.179, tau −0.115).

## Discussion

### Natural selection in octopus haemocyanin

Mollusc haemocyanin evolved from a α-subclass tyrosinase some 740 million years ago [2, 33] and has evolved independently of arthropod haemocyanin since this time [34, 35]. Cephalopod haemocyanin emerged about 520 million years ago [2] with modern coleoid cephalopods (e.g. octopods, squids and sepiids) separating from the ancient and sluggish nautiloids around 420 million years ago [2]. Since this time, the haemocyanin of coleoid cephalopods has evolved to keep pace with the increased metabolic demands imposed by an increasingly competitive environment, particularly since the emergence of the faster moving fishes [8–10]. Given the several hundreds of millions of years of evolutionary history, it is thus not surprising that the cephalopod haemocyanin gene underwent extensive purifying selection (Fig. 4) leading to widespread sequence conservation as has been confirmed for *Enteroctopus dofleini* [23]. High conservation of sites being directly involved in oxygen binding - such as the copper binding centre - indicate rather indirect mechanisms modulating oxygen binding in octopods.

Nevertheless, comparisons of two partial haemocyanin regions among octopods from various climates revealed evidence for natural selection acting on the Octopus haemocyanin gene. This is indicated by phylogenetic deviations between the mitochondrial genes and the two investigated haemocyanin regions (Figs. 1, 3) and also by the presence of variable sites within otherwise highly conserved regions (Fig. 4a), and lastly pinpointed by sites with increased rates of non-synonymous substitutions confirmed by various selection tests (Fig. 4b, c, Table 1). Unfortunately, studies assessing positive selection in mollusc haemocyanins are lacking and at least scarce in other groups. Positive selection was reported for lobster haemocyanins due to high non-synonymous substitution rates estimated for whole sequence regions in some species [36, 37], however analysis of single codon sites failed to confirm these findings [37]. This first report of positive selection in cephalopod haemocyanin indicates the presence of sites potentially involved in functional adaptation of haemocyanin.

### Structural links to protein function

Although *Octopus* haemocyanin contains sites identified to be positively selected one needs to assess their functional relevance. We thus analysed all 13 selected sites for their structural properties to deduce potential functional effects.

Our analyses showed that selection prevailed on residues located at the surface of the haemocyanin FU g and FU f, mostly affecting polar/charged properties (Fig. 5, Table 1), suggesting functional relevance for tertiary or quaternary interactions. Polar and particularly charged surface residues play a major role in stabilizing contacts among functional units of cephalopod haemocyanin via salt bridges [21]. Further, cooperativity, often found to be pronounced in cephalopods [38], has been proposed to operate via various interfaces among all seven FU types [21]. The partial regions analysed in this study are part of FU f and FU g, which interact with other FU via various surface interfaces. These comprise the closely spaced morphological unit interface FU c↔f, the horizontal tier interface FU e↔f and the arc wall interface FU g1/g2↔d as well as the more distant major groove interface FU a↔f and the arc morphological unit interface FU g1↔g2 characterized by weaker interactions [21]. Based on the detailed interface models of *Nautilus* haemocyanin [21], contact residues for the morphological unit interface FU c↔f were identified as F2393, 2428WHFDRT2433 and P2479 for the octopod haemocyanin sequences analysed in this study (corresponds to H2401, 2437WKYDRL2442 and H2488 in *Nautilus*). Interestingly, this region did not contain any of the variable and positively selected sites, and in fact, was highly conserved across all 113 sequences (Fig. 4a, Additional file 4). Therefore, the morphological unit interface FU c↔f seems a less likely target for direct functional regulation, although significant sequence differences of haemocyanins between octopods and *Nautilus* suggest further or different contact regions at this interface than proposed for *Nautilus* haemocyanin only. Contact residues for the alternative interfaces FU e↔f , FU g1/g2↔d, FU a↔f and FU g1↔g2 were located outside the analysed sequence region and remain to be assessed in subsequent studies.

Only three buried residues were identified to be positively selected, out of which residue 2545 has high potential to be an allosteric site. This is due to its linking position between the copper binding histidine (His2543) and the nearby FU surface as well as its immediate neighbourhood composed of hydrophobic and hydrophilic amino acids. These create a hydrophobic/hydrophilic contrast, which promotes metal binding, particularly in the presence of the sulphur carrying methionine [39–41]. A conformational movement of residue 2545 upon allosteric binding could easily transfer to the two amino acid distant copper binding His2543 and affect oxygen binding, as minor shifts of only 0.7 Å between the coordinated copper ions suffice to change oxygenation [21].

The remaining positively selected buried residues 2575 and 2442 are less likely to be involved in functional regulation. Despite the proximity of site 2575 to the copper binding His2571, neither alanine, valine nor threonine had the possibility to interact directly with the active site. Alanine and valine are very non-reactive [42] and were too distant to affect the active site. Direct polar interaction of threonine was also unlikely, as the only nearby polar group, the terminal amino group of His2571, was involved in a covalent peptide bond. Moreover, inward facing residues involved at site 2442 were exclusively hydrophobic (methionine, isoleucine, leucine or valine), non-reactive and due to their large distance to the active site unlikely to be involved in functional regulation.

Although variation and positive selection were most significant at site 2503 (Fig. 4), functional relevance is rather unlikely. Site 2503 is located at the beginning of the linker region connecting FU f and FU g1/g2 that, like all inter-FU linkers, comprise a long (34–57 Å), drawn-out sequence between 12–20 amino acids [21]. Given this high length and the capacity to extend further, as linkers are unlikely to extend to their maximum length [21], there is little chance that the identified substitutions among alanine, valine, threonine, or serine (Additional file 4: Figure S4) affect the positioning or conformation of the ca. 51 Å distant FU g1/g2.

### Correlates of temperature adaptation

Temperature affects oxygen supply [13], a challenge that octopods have been required to overcome in order to colonize oceans from the tropics to the poles, where they now thrive at high diversity and abundance [4]. Low temperatures increase the affinity of haemocyanin for oxygen [38, 43], thus hampering oxygen release to the tissue, to an extent that it limits oxygen supply in cephalopods [15, 16]. On the other hand, lower temperatures increase levels of physically dissolved oxygen in the blood/haemolymph [44, 45] and decrease mass specific metabolic demands for oxygen of ectothermic animals [46–48], which enabled a reduction or even a complete loss of haemoglobin in Antarctic notothenioid fishes [45, 49]. Antarctic octopods similarly benefit from relaxed oxygen requirements in the cold (e.g. metabolic rate of 0.319 mmol O_2_ kg^−1^ (wet mass) h^−1^ at 0 °C in the Antarctic *Pareledone charcoti* vs. 2.672 mmol O_2_ kg^−1^ at 21 °C in the subtropical *Octopus vulgaris* [48, 50] and dissolved oxygen contributing 18.5 % to total haemolymph oxygen content in *Pareledone charcoti* [16]). Nevertheless, Antarctic octopods continue to rely on active oxygen transport. This is marked by 39 % to 46 % higher concentrations of haemocyanin in the haemolymph and functional modifications of haemocyanin comprising a lower affinity for oxygen, increased rates of pH dependent oxygen release as well as a shift of the pH sensitive range of oxygen binding towards higher pH values compared to octopods from warmer waters [16].

So far, our data has highlighted positive selection acting on surface charges and a potential allosteric site. We thus assessed whether this explains differences among octopods from polar, temperate, subtropical or tropical climates and in particular the functional adaptations observed in cold-adapted Antarctic octopods [16]. The analysis of polar surface residues revealed a clear distinction between cold and warm adapted octopods dominated by a decrease of glutamic acid and an increase of positively charged amino acids towards colder climates, causing a higher net surface charge in polar octopods compared to octopods from warmer waters (Fig. 6). Importantly, the comparison between the Antarctic octopus *Pareledone charcoti* and the temperate *Enteroctopus dofleini* revealed that this pattern not only applies to the partial regions FU f-g and FU g but also to most of the entire haemocyanin gene (except FU a, Fig. 7). This indicates that climate dependent selection of surface charge properties occurred in parallel for several FUs, although full haemocyanin sequences of more and particularly tropical species need to be included to further substantiate these findings.

**Fig. 8:**
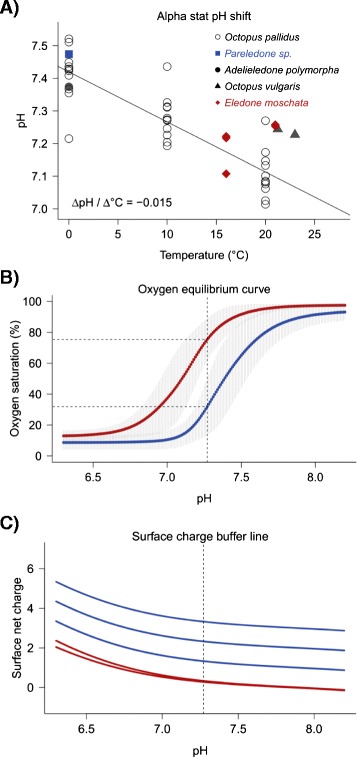
Functional and structural pH dependence. **a** pH change of octopod haemolymph following an imidazole like alpha stat pattern of −0.0153 pH units / °C (redrawn from [16]) **b**) Differences between the Antarctic *Pareledone charcoti* (blue) and the Mediterranean *Eledone moschata* (red) regarding pH dependent oxygen binding at 10 °C and 1kPa *P*O_2_ (shaded area denotes 95 % C.I., *n* = 5, data taken from [16]) and **c**) net surface charge of the partial haemocyanin region FU f-g at pH 7.27 based on six partial haemocyanin sequences for each species, whose buffer lines partly overlap. The dashed vertical line indicates the venous pH at 10 °C interpolated from **a**)

Changes of net surface charge properties help to explain how cold-adapted octopods attenuate the detrimental effect of increased oxygen affinity. Perutz [51] provided the first structural explanation for altered oxygen affinity in haemoglobin, based on a His-Asp salt bridge linking two haemoglobin subunits. High ambient pH deprotonates the histidine and disrupt this salt bridge, which destabilizes or dilates the haemoglobin quaternary structure and thus increases oxygen affinity by adjacent subunits. Haemocyanin may respond similarly. It not only dissociates into its subunits at higher pH [52, 53] but is also highly pH sensitive with Bohr coefficients well below −1 [26, 54]. Further, unlike intracellular haemoglobin [55], haemocyanin is not protected from changes of haemolymph pH. Consequently, one would expect even larger effects of pH disturbances on haemocyanin structure than on haemoglobin. Octopods living in polar waters face higher haemolymph pH than warm water species due to the temperature dependency of equilibrium constants (pK) of ionisable groups, particularly the imidazole groups of proteins (i.e. alpha stat pattern, Fig. 8a, [16, 56, 57]). Such high haemolymph pH contributes to increased oxygen affinity and to the observed impairment of venous oxygen release towards colder temperatures [15, 16]. As a result, an increase of oxygen affinity in response to a cold-induced pH increase may be reduced or prevented if haemocyanin quaternary structure remains stable despite higher pH. The disruption of salt bridges linking haemocyanin FUs upon pH changes, as they occur during temperature changes, depends on the pK values of their ionisable groups, which are not fixed but variable depending on their protein environment [58]. Charge-charge interactions at the protein surface are among the most important factors disturbing the pK of ionisable groups, as evidenced by increased numbers of positively charged lysine, which raise the pK value of several surface residues, by up to 2.19 units [58]. Experimental substitutions from glutamic acid to lysine also confirmed increased stability (1.1 kcal mol^−1^) due to charge-charge interactions at the surface of Ribonuclease T1 [59]. Therefore, considering a more positive net surface charge in polar octopods, due to increased numbers of positively charged amino acids and decreased numbers of the negatively charged glutamic acid (Fig. 6), charge-charge interactions are likely to raise pK of residues linking FU interfaces. Consequently, salt bridges withstand disturbance by high pH occurring at low temperatures and retain the stability of the haemocyanin quaternary structure and accordingly the affinity for oxygen. This is in good agreement with oxygen binding in the Antarctic octopod *Pareledone charcoti*, which showed a lower oxygen affinity than the subtropical octopod *Eledone moschata* at the same pH and temperature (10 °C [16]). This compensation of oxygen affinity was particularly due to a shift of the pH dependent oxygen binding range in *Pareledone charcoti* towards higher pH (Fig. 8b, [16]). Theoretical buffer lines of surface residues of the partial haemocyanin sequence FU f-g confirm the more positive net surface charge in *Pareledone charcoti* compared to *Eledone moschata* at a venous pH of 7.27 (Fig. 8c). Altered surface charge properties were also suggested to facilitate oxygen secretion of haemoglobin (i.e. Bohr and Root effect) via a decrease of non-conserved histidine residues, which lower the capacity to buffer pH [60]. Similarly, myoglobin underwent changes of net surface charge to increase oxygen storage capacity in diving mammals via increased electrostatic repulsion, which reduces self-association of myoglobin [61]. However, unlike gastropod haemocyanin [22, 62], cephalopod haemocyanin does not assemble to multi-decameric complexes [63]. Thus electrostatic interactions likely occur intrinsically among the haemocyanin FUs.

Further, the proposed charge-charge interactions at the surface of FU g and FU f modulate oxygen binding rather via indirect modifications. This is underpinned by the high conservation of the morphological unit interface FU c↔f and the more uniform distribution of positively selected residues at the protein surface.

The presence of two very distinct isoforms in FU g, which does not follow the observed correlation between net surface charge and climatic origin, also supports the view of functionally divergent isoforms that are regulated via differential expression to enable flexible responses to changing environmental or metabolic conditions [64, 65]. Indeed, native page gel electrophoresis showed differential expression of haemocyanin isoforms in the Antarctic squid *Moroteuthis ingens* (Oellermann et al. unpublished). However, this could not be documented for octopod haemocyanin so far as their isoforms rarely separate on native gels, except for the Antarctic octopod *Pareledone turqueti*, which showed a relatively even expression between two distinct haemocyanin isoforms (Oellermann et al. unpublished). Similarly, in the cuttlefish *Sepia officinalis* isoforms levels of haemocyanin mRNA do not differ in adult specimens or in response to temperature or hypercapnia and change only during ontogeny [25]. Therefore it remains unclear if octopods employ differential haemocyanin expression to modulate oxygen binding and if polar octopods express their “high net surface charge” isoform of FU g (Fig. 6b) at significant levels to modulate oxygen binding.

Distinct evolutionary histories of the two Antarctic octopod families may explain why the Enteroctopodidae did not share the same pattern of charged surface residues as the Megaleledonidae (Fig. 6). The Megaleledonidae are thought to have had their evolutionary origins in Antarctica before the onset of glaciation around 50 Ma years ago [66] whereas the Enteroctopodidae are thought to have had their evolutionary origins in shallow Northern Hemisphere waters from where they moved in to the deep sea and subsequently to Antarctic waters [27, 67]. Therefore, Enteroctopodidae may have followed a diverging strategy to cope with cold temperatures either marked by a total lack of compensation or via physiological adjustments that did not involve haemocyanin. Such alternative adjustments may comprise increased blood circulation or ventilation, as indicated in *Benthoctopus spp.* (Enteroctopodidae) that shows higher metabolic enzyme activities in mantle muscle than *Graneledone boreopacificus* (Megaleledonidae), which shares the same deep sea habitat [68].

Moreover, residue 2545 has the potential as an allosteric site and interestingly contained mostly methionine in polar octopods and mostly leucine in tropical octopods. The sulphur group of methionine plays an important role in coordinating cations such as copper ions in other “blue” proteins such as plastocyanins or azurins [40] as well as in copper trafficking protein [69, 70]. In fungal laccases, substitution of methionine with non-coordinating leucine or phenylalanine breaks a copper-methionine interaction, likely leading to significant changes of the redox potential at this site [40, 71]. Experimental substitution of methionine with norleucine further confirmed specific binding of copper by the sulphur group of methionine [72]. Although methionine was most frequently found to bind copper, it is also able to form stable complexes with magnesium [41], indicating a general affinity for divalent cations. Therefore, a hydrophilic/hydrophobic contrast [39] together with a selective preference of methionine in polar and leucine in warm water octopods at site 2545 may serve to regulate oxygen affinity in response to temperature adaptation via altered affinity to allosteric metals. Such intrinsic regulation would provide a reasonable mechanism to exploit for example magnesium as oxygen affinity regulator despite the octopods’ inability to regulate extracellular magnesium concentrations [16].

## Conclusions

This study provided the first insights into molecular and structural mechanisms that enabled octopods to sustain oxygen supply at sub-zero temperatures. In particular, we revealed natural selection acting on two partial gene regions of the octopods’ oxygen transporter haemocyanin. Predominant selection of charge properties at the surface of haemocyanin FU g and FU f as well as a more positive net surface charge of the entire haemocyanin gene of the Antarctic octopus *Pareledone charcoti* indicated modulation of oxygen binding behaviour via charge-charge interaction. A more positive net surface charge in cold-adapted octopods is suggested to stabilize quaternary structure to sustain a lower oxygen affinity under a high pH environment. Selection of a residue linking the surface with the active site further indicates modulation of oxygen affinity via increased sensitivity to allosteric metal binding in cold-adapted octopods. Given the high conservation of numerous functional sites being directly involved in oxygen binding or quaternary interactions, modulation of oxygen binding in octopods may prevail on indirect mechanisms such as altered surface charge properties.

Future studies may further assess the mechanisms pinpointed in this study by means of obtaining entire haemocyanin sequences from more species to enable comparative modelling of quaternary interactions, ideally complemented by combined functional and mutational experiments on haemocyanin.

## Methods

### Study design

To assess whether natural selection and adaptation to particular climates acted on the evolution of cephalopod haemocyanin, we performed a comparative analysis using benthic octopods, an ideal study group not only because of their accessibility and similar physiological constitution but particularly because of their diverse presence in all major climates, which strongly facilitates analysis of temperature adaptation. We therefore collected samples of 28 octopod species originating from polar, temperate, subtropical and tropical habitats with species represented in each of the five recognised benthic incirrate octopod families (e.g. Eledonidae, Octopodidae, Enteroctopodidae, Megaleledonidae and Bathypolypodidae [27]). We did not sequence the full haemocyanin genes for all species to compromise between a high number of species and costs involved in sequencing multiple isoforms, each being more than 9000 bp long. We therefore constrained our main analysis to a partial coding region of the FU f to represent FUs (a-f), which form the wall structure of the cylindrical haemocyanin decamer, and a partial coding region of the FU g, which forms a relatively loosely connected collar structure inside the cylinder [21]. Due to their distinct quaternary arrangement, functional regulation and thus selection may differ.

### Sample acquisition

Samples of octopods were provided by collaborators or purchased from traders except for Antarctic octopods, which were collected during RV Polarstern cruises ANTXV-3, ANTXXVII-3 and ANTXXIII-4 (see http://expedition.awi.de for cruise- and Addtional file 2 & Additional file 3 for sample details).

Antarctic octopods were caught using bottom and Agassiz trawls and kept in temperature controlled aquaria until sampling. Prior to sampling, animals were anaesthetized in 3 % ethanol [73] until non-responsive, killed by a final cut through the brain and then opened ventrally for organ removal. Excised organs were immediately frozen in liquid nitrogen or preserved in RNAlater (QIAGEN, Germany) and stored at −80 °C.

Any handling and sampling of octopods complied with common ethical and experimental procedures for cephalopods [74] and was registered at the veterinary inspection office, Bremen, Germany (reg. no. 522-27-11/02- 370 00(93)). At the time of sampling, German and EU regulations did not require ethical approval for cephalopods [75]. Collection of Antarctic octopods complied with the general guidelines under §1 Umweltschutzprotokolle zum Antarktisvertrag (AUG).

### PCR, cloning and sequencing

Genomic DNA was extracted from gill glands, mantle tissue or arm tips using the QIAGEN DNeasy Blood and Tissue kit following the manufacturer’s instructions. To construct a species phylogeny for the sampled octopods, we amplified partial sequences of cytochrome c oxidase subunit I (COI) and cytochrome c oxidase subunit III (COIII), which are considered selectively neutral, using polymerase chain reaction (PCR) and the primers detailed in [76–78]. COI and COIII were amplified in 25 μl PCR mix containing final concentrations of 0.5 μmol L^−1^ dNTPs, 0.05 units μl^−1^ Taq DNA Polymerase, 1 x Taq buffer (5 Prime, Germany) and 1 μmol L^−1^ of each Primer. The PCR reaction comprised an initial denaturation at 94 °C for 4 mins, followed by 35 cycles at 94 °C for 40 s, 50 °C (COI) or 42 °C (COIII) respectively for 40 s, 68 °C for 90 s and a final extension step at 68 °C for 10 min.

Regions of the haemocyanin gene were amplified using two pairs of degenerate primers, which bind to conserved sites present across all seven functional units (i.e. amino acid sequence PYWDW and WAIWQ, [79]). Template specificity was enhanced via Touchdown-PCR in 25 μl reaction volume containing final concentrations of 0.2 μmol L^−1^ dNTPs, 0.05 units μl^−1^ DreamTaq DNA Polymerase (Thermo Scientific, Germany), 1 x DreamTaq Green buffer (Thermo Scientific, Germany) and 1 μmol L^−1^ of each primer. The PCR reaction comprised an initial denaturation at 94 °C for 4 mins, 12 cycles at 94 °C for 45 s, 60→48°C for 60 s (−1 °C/cycle), 72 °C for 90 s followed by 35 cycles at 94 °C for 45 s, 52 °C for 60 s, 72 °C for 90 s and a final extension at 72 °C for 8 min. PCR products were separated on 1.3 % agarose gel containing GelRed (Biotium, U.S.A.) and distinct bands excised and purified using the QIAQuick Gel Extraction Kit (QIAGEN, Germany). Purified PCR fragments were then cloned using the pGEM-T Easy vector system (Promega, Germany) due to the potential binding of the primer pairs at each of the seven FUs, the presence of isoforms as well as alleles. Plasmids of positive clones were purified using the QIAprep Spin Miniprep Kit (QIAGEN, Germany) and tested for successful insertion via an EcoRI (Life Technologies, Germany) restriction digest. Sanger sequencing of products was performed by Eurofins MWG Operon or GATC Biotech AG, Germany. Amplicons were most frequent for the regions FU f-g and FU g comprising fragments of 370 bp or 1090 bp length (Fig. 2). Amplicons of other regions were not represented across all sampled species and were thus not included in subsequent analysis.

In addition, we sequenced the entire haemocyanin gene of the Antarctic octopus *Pareledone charcoti*. First, RNA was extracted from gill glands using the QIAGEN RNeasy Mini Kit and transcribed to cDNA using SuperScript III Reverse Transcriptase (Invitrogen, Germany) and a custom 3’-end T-rich primer to enrich haemocyanin transcripts. Using the same set of degenerate primers as for partial sequencing, initial sequences were obtained using the protocol described above and subsequently sequence gaps closed by primer walking. 5’ and 3’ ends were sequenced using the First Choice RLM-RACE Kit (Ambion, Germany) according to the manufacturer’s instructions. Following agarose gel electrophoresis target fragments were excised, purified using the QIAQuick Gel Extraction Kit (QIAGEN, Germany) and submitted for Sanger sequencing (Eurofins MWG Operon or GATC Biotech AG, Germany). Sequence editing and assembly was performed using Geneious 7.1.5 (Biomatters, New Zealand).

### Phylogenetic analysis

Obtained sequences were assembled and verified by means of their chromatograms and primer sequences were trimmed using Geneious 7.1.5 (Biomatters, New Zealand). Published sequences further supplemented the COI (*Enteroctopus dofleini* [GenBank: GU802397], *Nautilus pompilius* [GenBank: AF120628]), the COIII (*Enteroctopus dofleini* [GenBank: X83103]) and the haemocyanin data sets (*Enteroctopus dofleini* [GenBank: AF338426 & AF020548]). COI and COIII were concatenated and multiple sequence alignments obtained using the MUSCLE plugin of Geneious [80]. The two amplified haemocyanin regions FU f-g and FU g were not concatenated as the presence of multiple isoforms as well as allelic variation did not allow reliable sequence matching. Thus, multiple sequence alignments and subsequent analysis were performed separately for each of the two regions. The intron region between the FU f and FU g was identified by means of the *Enteroctopus dofleini* haemocyanin sequence and trimmed from the alignment. For haemocyanin alignments, 3’ and 5’ ends were trimmed to obtain a consistent reading frame as well as gap free 3’ and 5’ ends, which would otherwise have produced false results in the selection analysis. Translated sequences containing stop codons were removed. Full haemocyanin sequences comprising two isoforms of FU a-g for *Enteroctopus dofleini* and one isoforms of FU a-g for *Pareledone charcoti* were aligned using MUSCLE and trimmed by hand. The quality of the COI/COII and haemocyanin alignments were tested using GBlocks 0.91b [81, 82] tolerating gap positions within final blocks, which retained between 98-100 % of the original alignment. Based on the Akaike Information Criteria [83], JModeltest 2.1.5 [84] identified the GTR + I + G model for the COI-COIII data set, the HKY85 model for the two haemocyanin regions and the Dayhoff model for the entire haemocyanin gene as the best available substitution models.

Based on the COI-COIII and haemocyanin alignments phylogenetic relationships were inferred using Bayesian and maximum likelihood methods. Bayesian trees were constructed using MrBayes [85] as implemented in Geneious (v. 2.0.3) running at least two independent Monte Carlo Markov Chain (MCMC) analysis with 10,000,000 generations sampled every 10,000 generation. The appropriate burnin was chosen based on the resulting traces, which showed a stationary distribution before 10 % of the MCMC chain. Maximum likelihood trees were constructed using the PhyML [86] plugin of Geneious and bootstrap values calculated from 1000 replicates. *Nautilus pompilius* and *Vampyroteuthis infernalis* were used as outgroups for the COI-COIII phylogeny.

### Selection analysis

Prior to selection analysis, we screened both partial haemocyanin regions for conserved and variable sites using the Jensen-Shannon Divergence [30], which identifies conserved sites as deviations of a probability distribution from the overall amino acid distribution of the respective BLOSUM62 alignment as background and also accounts for conservation in neighbouring sites.

To assess whether natural selection affected the evolution of octopod haemocyanin we employed codon-based Bayesian and maximum likelihood approaches to estimate rates of non-synonymous (*d*N) to synonymous substitutions (*d*S). Ratios ≤ 1 denote purifying or negative selection and ratios > 1 diversifying or positive selection. The unrooted haemocyanin phylogenies, without outgroup (Fig. 3a, b) were uploaded to the Datamonkey webserver [87, 88] and selection inferred via the following methods. Prior to performing selection tests, alignments were tested for recombination using Genetic Algorithms for Recombination Detection analysis (GARD) implemented in Datamonkey. Single sites under selection were identified using Single Likelihood Ancestral Counting (SLAC), Fixed Effects Likelihood (FEL), Mixed Effects Model of Evolution (MEME), Fast Unconstrained Bayesian AppRoximation (FUBAR), Evolutionary Fingerprinting (EF) as well as PRoperty Informed Models of Evolution (PRIME). SLAC estimates and compares normalized expected and observed numbers of synonymous and non-synonymous substitutions at each codon position based on a single ancestral sequence reconstruction [89]. FEL estimates and compares *d*N and *d*S independently for each site [89]. MEME assesses whether single sites undergo positive as well as episodic diversifying selection along particular branches [90]. FUBAR enables larger numbers of site classes and efficiently identifies positively selected sites using a hierarchical Bayesian MCMC routine [91]. EF compares ‘evolutionary fingerprints’ between homologous as well as non-homologous sequences obtained from a posterior sample of a bivariate distribution of *d*N and *d*S at each site [92]. PRIME resembles FEL or MEME but additionally links an amino acid property category [93, 94] to the non-synonymous substitution rate. Significance thresholds for selection tests were: *P* ≤ 0.10 for SLAC, FEL and MEME; *P* ≤ 0.05 for PRIME; posterior probability ≥ 0.90 for FUBAR and Bayes factor ≥ 0.50 for EF.

We further employed the software TreeSAAP 3.2 (Selection on Amino Acid Properties using phylogenetic trees, [95]) to analyse which out of 31 amino acid properties are under positive selection. TreeSAAP categorizes these physico-chemical properties into 8 magnitudes with low magnitudes being more conservative and high magnitudes being more radical and assesses codon by codon whether the distribution of observed changes of amino acid properties differs from an expected uniform distribution. We considered changes of amino acid properties of codons with magnitudes ≥ 6 and z-scores ≤ 0.001 to be positively selected. In this study, sites were considered positively selected if at least three tests yielded significant results.

Amino acid sites under selection were illustrated with PhyMol 1.3 [96] using the crystal structure of the functional unit g based and the protein sequence of *Enteroctopus dofleini* ([PDB: 1JS8], [23, 24]). The homologous partial haemocyanin region of FU f was aligned with the FU g PDB sequence to match and display positively selected FU f residues on the 3D protein structure.

### Analysis of polar surface residues

Polar surface residues were assessed for differences between octopods originating from different climates. First, surface residues of the FU g crystal structure were identified via the GETAREA webserver [97], setting the radius of the water probe to 1.4. This yielded 65 surface residues for the partial haemocyanin fragment FU f-g, 19 surface residues for the partial haemocyanin fragment FU g and 85 surface residues for each of the FUs of the full haemocyanin sequences. Numbers of each type of polar surface residues as well as net charge (at pH 7.27) were determined and in case of the partial regions FU f-g and FU g analysed via principal component analysis to assess correlation patterns and the impact of climatic origin.

Sequence processing, statistical analysis and graphical display were performed with the ‘R’ statistical language [98] and the packages ‘seqinr’ [99], ‘ape’ [100] and ‘ade4’ [101] if not mentioned otherwise.

### Availability of supporting data

Sequences, alignment and tree files can be found in Additional file 1 and are also available at PANGAEA® - Data Publisher for Earth & Environmental Science [102]. In addition, COI barcode sequences, photos and geolocations of sampled octopods can be accessed from Barcode of Life Data Systems (BOLDSystems).

## Additional files

Additional file 1: Tree and alignment files of haemocyanin, COI and COIII sequences used in this study provided in nexus format. Additional file 2. Location and sampling details of octopods used in this study. Additional file 3: Mapped sample locations of octopods used in this study. Additional file 4: Multiple protein sequence alignment of octopus haemocyanin covering A) a 286 and B) a 110 amino acid long region across the functional units F and G. Sequences represent homologous copies and isoforms of this partial haemocyanin region from within individuals as well as multiple individuals of various octopus species. Additional file 5: Detailed results of selection tests.

## Abbreviations

FU: Functional unit
ML: Maximum likelihood
COI: Cytochrome c oxidase subunit I
COIII: Cytochrome c oxidase subunit III
JSD: Jensen-shannon divergence
*d*N: Rate of non-synonymous codon substitutions
*d*S: Rate of synonymous codon substitutions
PCR: Polymerase chain reaction
GARD: Genetic algorithm for recombination detection
SLAC: Single likelihood ancestral Counting
FEL: Fixed effects likelihood
MEME: Mixed effects model of evolution
FUBAR: Fast unconstrained bayesian approximation
EF: Evolutionary fingerprinting
PRIME: PRoperty informed models of evolution
