# Erratum to: Positive selection in octopus haemocyanin indicates functional links to temperature adaptation

**DOI:** 10.1186/s12862-015-0536-5

**Published:** 2015-12-02

**Authors:** Michael Oellermann, Jan M. Strugnell, Bernhard Lieb, Felix C. Mark

**Affiliations:** Integrative Ecophysiology, Alfred-Wegener-Institute Helmholtz Centre for Polar and Marine Research, Am Handelshafen 12, Bremerhaven, 27570 Germany; Department of Genetics, La Trobe Institute for Molecular Sciences, La Trobe University, Bundoora, 3086 VIC Australia; Institute of Zoology, Johannes Gutenberg-Universität, Müllerweg 6, Mainz, 55099 Germany

## Erratum

The original version of this article [1] unfortunately contained a mistake. The presentation of Table 1 along with the table legend and footnote was incorrect in the HTML and PDF versions of this article. The corrected version is given below.

**Table 1:** Positively selected sites in octopus haemocyanin at which at least three or more selection tests identified significant positive selection. Analysis was performed for two separate alignments, containing 113 or 126 sequences respectively, and covering in total a 396 amino acid long region of the haemocyanin’s functional units f and g. Numbering of positions refers to the published full haemocyanin sequence of *Enteroctopus dofleini* [UniProt: O61363] [23, 24]. Significance thresholds were: P ≤ 0.10 for SLAC, ≤ 0.10 for FEL, MEME and PRIME; Posterior Probability ≥ 0.90 for FUBAR; Bayes Factor ≥ 0.50 for EF. See Additional file 5 for detailed results

Residue	SLAC^a^	FEL^a^	MEME^a^	FUBAR^a^	EF^a^	PRIME^b^				TreeSAAP^c^			
										pHi	pK’	pα	PCT
2383		*	*								↓(1) ↑(2–4)		
2409	*	*	*	*	*								
2410	*	*	*								↓(5–8)		
2442					*	(CC)					↓(10) ↑(5,9,11)		
2469			*			CC						↓(12,13)	
2496		*	*	*	*					↓(14,15)			
2503	*	*	*	*	*	(P)						↓(14,15,22–27) ↑(20)	
2545				*	*						↓(4, 20, 28–30) ↑(9,19,31,32)		
2575			*		*							↓(33,34)	
2585		*	*	*	*	pHi	CC	(P)	(V)				
2602		*	*							↓(35,36)			↑(35,36)
2610		*	*			pHi				↓(37–39) ↑(40,41)			↓(40–42) ↑(37–39)
2643			*			CC							↓(38)

Abbreviations: *CC* chemical composition, *P* polarity, *V* volume, *pHi* iso-electric point, *pK’* equilibrium constant (ionization of COOH), *pα* alpha helical tendencies, *PCT* power to be at the C-terminal

^a^Sites at which SLAC, FEL, MEME, FUBAR or EF identified positive selection are indicated with asterisks

^b^Sites at which positive selection was inferred with PRIME. Properties under selection are indicated in bold letters and properties being conserved are indicated in parenthesis

^c^Sites at which positive selection was inferred with TreeSAAP. Magnitude of amino acid change was 8 for pHi and pK´ and 6 for pα and PCT. Arrows indicate an increase (↓) or decrease (↑) of the amino acid property. Parenthesis mark the affected branches shown in Figure 3
