# Supplementary material for: Positive selection in octopus haemocyanin indicates functional links to temperature adaptation
Source: BMC Evol Biol. 2015 Jul 5;15:133. doi: 10.1186/s12862-015-0411-4 (PMC4491423; doi:10.1186/s12862-015-0411-4)
Supplement: Additional file 3: — Mapped sample locations of octopods used in this study. [file 12862_2015_411_MOESM3_ESM.pdf]

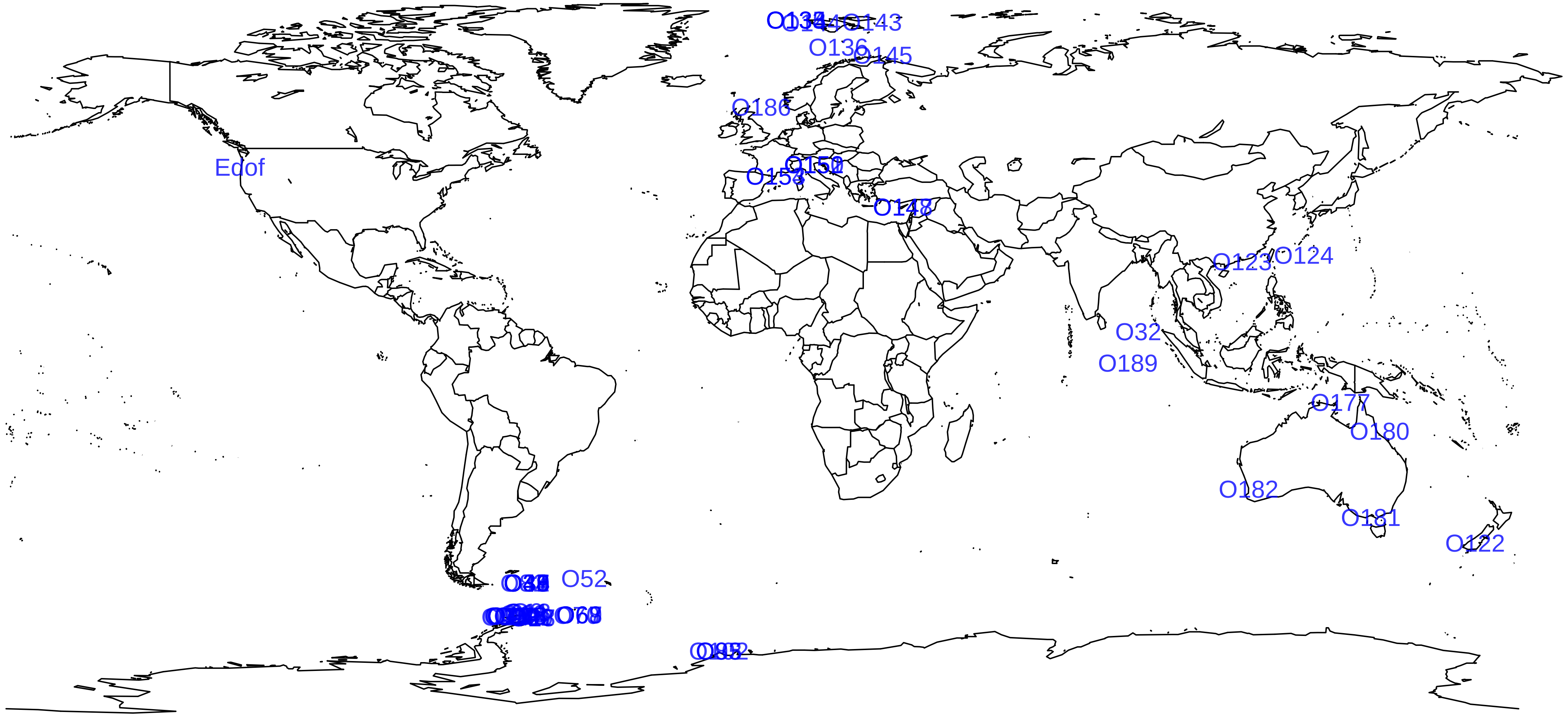


*O85 Adelieledone polymorpha*
*O32 Amphioctopus aegina*
*O143 Bathypolypus arcticus*
*O144 Bathypolypus arcticus*
*O133 Bathypolypus pugniger*
*O134 Bathypolypus pugniger*
*O135 Bathypolypus pugniger*
*O136 Bathypolypus pugniger*
*O145 Bathypolypus pugniger*
*O39 Benthoctopus cf. longibrachus*
*O52 Benthoctopus cf. longibrachus*
*O41 Benthoctopus cf. rigbyae*
*O44 Benthoctopus cf. rigbyae*
*O45 Benthoctopus cf. rigbyae*
*O48 Benthoctopus cf. rigbyae*
*O49 Benthoctopus cf. rigbyae*
*O82 Benthoctopus sp.*
*O148 Callistoctopus macropus*
*O180 Callistoctopus ornatus*
*O186 Eledone cirrhosa*
*O147 Eledone moschata*
*O150 Eledone moschata*
*O151 Eledone moschata*
*O152 Eledone moschata*
*Edof Enteroctopus dofleini*
*O36 Graneledone yamana*
*O37 Graneledone yamana*
*O177 Hapalochlaena lunulata*
*O181 Hapalochlaena maculosa*
*O122 Macroctopus maorum*
*O11 Megaleledone setebos*
*O19 Megaleledone setebos*
*O9 Megaleledone setebos*
*O189 Octopus membranaceus*
*O182 Octopus tetricus*
*O123 Octopus vulgaris*
*O124 Octopus vulgaris*
*O153 Octopus vulgaris*
*O154 Octopus vulgaris*
*O27 Pareledone aequipillae*
*O28 Pareledone aequipillae*
*O31 Pareledone aequipillae*
*O13 Pareledone aurata*
*O68 Pareledone aurata*
*O118 Pareledone charcoti*
*O155 Pareledone charcoti*
*O158 Pareledone charcoti*
*O69 Pareledone cornuta*
*O70 Pareledone cornuta*
*O22 Pareledone felix*
*O74 Pareledone felix*
*O80 Pareledone felix*
*O67 Pareledone panchroma*
*O102 Pareledone prydzensis*
*O93 Pareledone prydzensis*
*O20 Pareledone turqueti*
*O30 Pareledone turqueti*
*O16 Pareledone turqueti*
