# Supplementary material for: Positive selection in octopus haemocyanin indicates functional links to temperature adaptation
Source: BMC Evol Biol. 2015 Jul 5;15:133. doi: 10.1186/s12862-015-0411-4 (PMC4491423; doi:10.1186/s12862-015-0411-4)
Supplement: Additional file 4: — Multiple protein sequence alignment of octopus haemocyanin covering A) a 286 and B) a 110 amino acid long region across the functional units F and G. Sequences represent homologous copies and isoforms of this partial haemocyanin region from within individuals as well as multiple individuals of various octopus species. [file 12862_2015_411_MOESM4_ESM.pdf]

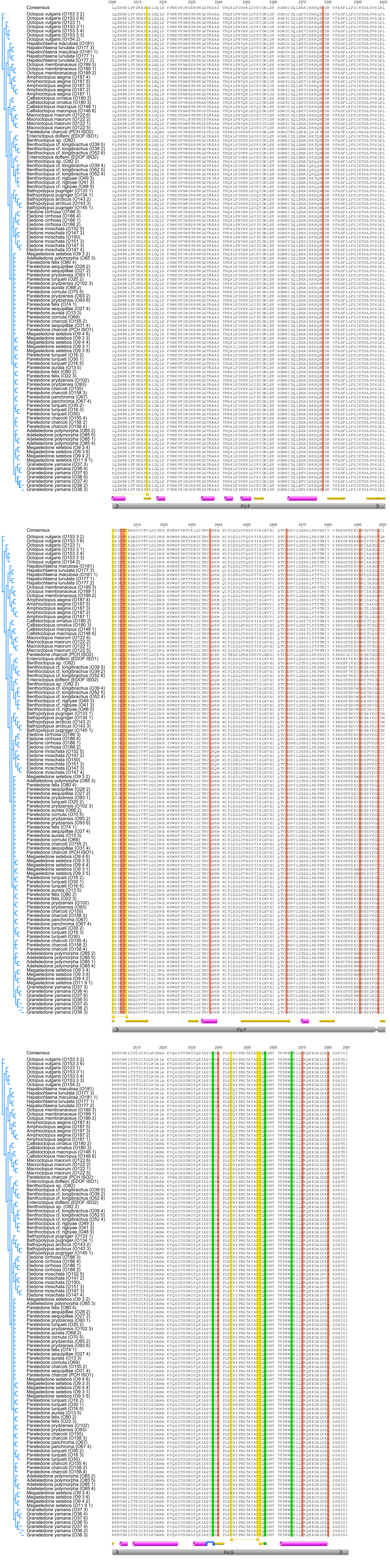
Consensus
FU F
Consensus
FU F
Consensus
FU G

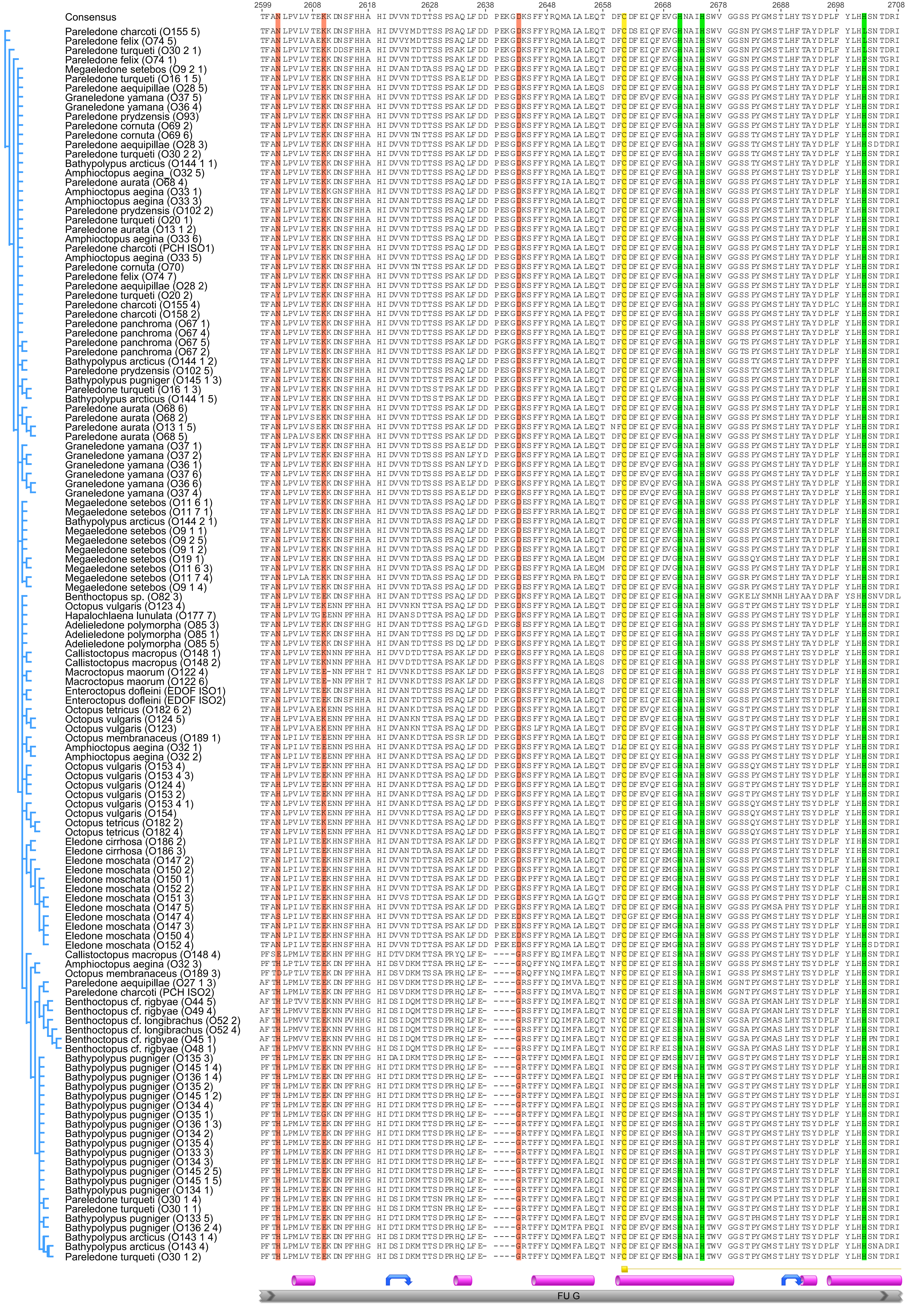

2599 2608 2618 2628 2638 2648 2658 2668 2678 2688 2698 2708
Consensus
Pareledone charcoti (O155 5)
Pareledone felix (O74 5)
Pareledone turqueti (O30 2 1)
Pareledone felix (O74 1)
Megaeledone setebos (O9 2 1)
Pareledone turqueti (O16 1 5)
Pareledone aequipillae (O28 5)
Graneledone yamana (O37 5)
Graneledone yamana (O36 4)
Pareledone prydzensis (O93)
Pareledone cornuta (O69 2)
Pareledone cornuta (O69 6)
Pareledone aequipillae (O28 3)
Pareledone turqueti (O30 2 2)
Bathypolypus arcticus (O144 1 1)
Amphioctopus aegina (O32 5)
Pareledone aurata (O68 4)
Amphioctopus aegina (O33 1)
Amphioctopus aegina (O33 3)
Pareledone prydzensis (O102 2)
Pareledone turqueti (O20 1)
Pareledone aurata (O13 1 2)
Amphioctopus aegina (O33 6)
Pareledone charcoti (PCH ISO1)
Amphioctopus aegina (O33 5)
Pareledone cornuta (O70)
Pareledone felix (O74 7)
Pareledone aequipillae (O28 2)
Pareledone turqueti (O20 2)
Pareledone charcoti (O155 4)
Pareledone charcoti (O158 2)
Pareledone panchroma (O67 1)
Pareledone panchroma (O67 4)
Pareledone panchroma (O67 5)
Pareledone panchroma (O67 2)
Bathypolypus arcticus (O144 1 2)
Pareledone prydzensis (O102 5)
Bathypolypus pugniger (O145 1 3)
Pareledone turqueti (O16 1 3)
Bathypolypus arcticus (O144 1 5)
Pareledone aurata (O68 6)
Pareledone aurata (O68 2)
Pareledone aurata (O13 1 5)
Pareledone aurata (O68 5)
Graneledone yamana (O37 1)
Graneledone yamana (O37 2)
Graneledone yamana (O36 1)
Graneledone yamana (O37 6)
Graneledone yamana (O36 6)
Graneledone yamana (O37 4)
Megaeledone setebos (O11 6 1)
Megaeledone setebos (O11 7 1)
Bathypolypus arcticus (O144 2 1)
Megaeledone setebos (O9 1 1)
Megaeledone setebos (O9 2 5)
Megaeledone setebos (O9 1 2)
Megaeledone setebos (O19 1)
Megaeledone setebos (O11 6 3)
Megaeledone setebos (O11 7 4)
Megaeledone setebos (O9 1 4)
Benthoctopus sp. (O82 3)
Octopus vulgaris (O123 4)
Hapalochlaena lunulata (O177 7)
Adelieledone polymorpha (O85 3)
Adelieledone polymorpha (O85 1)
Adelieledone polymorpha (O85 5)
Callistoctopus macropus (O148 1)
Callistoctopus macropus (O148 2)
Macroctopus maorum (O122 4)
Macroctopus maorum (O122 6)
Enteroctopus dofleini (EDOF ISO1)
Enteroctopus dofleini (EDOF ISO2)
Octopus tetricus (O182 6 2)
Octopus vulgaris (O124 5)
Octopus vulgaris (O123)
Octopus membranaceus (O189 1)
Amphioctopus aegina (O32 1)
Amphioctopus aegina (O32 2)
Octopus vulgaris (O153 4)
Octopus vulgaris (O153 4 3)
Octopus vulgaris (O124 4)
Octopus vulgaris (O153 2)
Octopus vulgaris (O153 4 1)
Octopus vulgaris (O154)
Octopus tetricus (O182 2)
Octopus tetricus (O182 4)
Eledone cirrhosa (O186 2)
Eledone cirrhosa (O186 3)
Eledone moschata (O147 2)
Eledone moschata (O150 2)
Eledone moschata (O150 1)
Eledone moschata (O152 2)
Eledone moschata (O151 3)
Eledone moschata (O147 5)
Eledone moschata (O147 4)
Eledone moschata (O147 3)
Eledone moschata (O150 4)
Eledone moschata (O152 4)
Callistoctopus macropus (O148 4)
Amphioctopus aegina (O32 3)
Octopus membranaceus (O189 3)
Pareledone aequipillae (O27 1 3)
Pareledone charcoti (PCH ISO2)
Benthoctopus cf. rigbyae (O44 5)
Benthoctopus cf. rigbyae (O49 4)
Benthoctopus cf. longibrachus (O52 2)
Benthoctopus cf. longibrachus (O52 4)
Benthoctopus cf. rigbyae (O45 1)
Benthoctopus cf. rigbyae (O48 1)
Bathypolypus pugniger (O135 3)
Bathypolypus pugniger (O145 1 4)
Bathypolypus pugniger (O136 1 4)
Bathypolypus pugniger (O135 2)
Bathypolypus pugniger (O145 1 2)
Bathypolypus pugniger (O134 4)
Bathypolypus pugniger (O135 1)
Bathypolypus pugniger (O136 1 3)
Bathypolypus pugniger (O134 2)
Bathypolypus pugniger (O135 4)
Bathypolypus pugniger (O133 3)
Bathypolypus pugniger (O134 3)
Bathypolypus pugniger (O145 2 5)
Bathypolypus pugniger (O145 1 5)
Bathypolypus pugniger (O134 1)
Pareledone turqueti (O30 1 4)
Pareledone turqueti (O30 1 1)
Bathypolypus pugniger (O133 5)
Bathypolypus pugniger (O136 2 4)
Bathypolypus arcticus (O143 1 4)
Bathypolypus arcticus (O143 4)
Pareledone turqueti (O30 1 2)
FU G

α-helix

β-strand

Turn

Copper ligand histidine

Disulfide bridge

Thioether bridge

Positively selected site
